# Effect of mesenchymal stromal cell infusions on lung function in COPD patients with high CRP levels

**DOI:** 10.1186/s12931-021-01734-8

**Published:** 2021-05-08

**Authors:** Daniel J. Weiss, Karen Segal, Richard Casaburi, Jack Hayes, Donald Tashkin

**Affiliations:** 1 University of Vermont College of Medicine, 226 Health Science Research Facility, Burlington, VT 05405 USA; 2 SSI Strategy New York, New York, NY USA; 3 Lundquist Institute for Biomedical Innovation at Harbor-UCLA Medical Center, Torrance, CA USA; 4 Mesoblast, Inc., New York, NY USA; 5 UCLA David Geffen School of Medicine, Los Angeles, CA USA

**Keywords:** Mesenchymal stromal cells, Chronic obstructive pulmonary disease, Inflammation, Pulmonary function, C-reactive protein

## Abstract

**Background:**

We previously reported a Phase 1/2 randomized placebo-controlled trial of systemic administration of bone marrow-derived allogeneic MSCs (remestemcel-L) in COPD. While safety profile was good, no functional efficacy was observed. However, in view of growing recognition of effects of inflammatory environments on MSC actions we conducted a post-hoc analysis with stratification by baseline levels of a circulating inflammatory marker, C-reactive protein (CRP) to determine the effects of MSC administration in COPD patients with varying circulating CRP levels.

**Methods:**

Time course of lung function, exercise performance, patient reported responses, and exacerbation frequency following four monthly infusions of remestemcel-L vs. placebo were re-assessed in subgroups based on baseline circulating CRP levels.

**Results:**

In COPD patients with baseline CRP ≥ 4 mg/L, compared to COPD patients receiving placebo (N = 17), those treated with remestemcel-L (N = 12), demonstrated significant improvements from baseline in forced expiratory volume in one second, forced vital capacity, and six minute walk distance at 120 days with treatment differences evident as early as 10 days after the first infusion. Significant although smaller benefits were also detected in those with CRP levels ≥ 2 or ≥ 3 mg/L. These improvements persisted variably over the 2-year observational period. No significant benefits were observed in patient reported responses or number of COPD exacerbations between treatment groups.

**Conclusion:**

In an inflammatory environment, defined by elevated circulating CRP, remestemcel-L administration yielded at least transient meaningful pulmonary and functional improvements. These findings warrant further investigation of potential MSC-based therapies in COPD and other inflammatory pulmonary diseases.

*Trial registration:* Clinicaltrials.gov NCT00683722.

## Background

Chronic obstructive pulmonary disease (COPD) is currently the fourth-ranked overall cause of death globally, with more than 120,000 attributable deaths annually in the United States [1]. COPD is characterized by a spectrum of clinical and pathophysiologic manifestations, but common features include chronic airway inflammation and progressive destruction of lung parenchyma. Currently, apart from lung transplantation, there are no curative treatments and available therapies are geared towards symptomatic relief. Owing to the high prevalence and chronic nature of the disease, COPD management has high resource utilization with frequent clinician visits, hospitalizations due to acute exacerbations, and requirement of chronic oxygen and pharmacologic therapies [1].

Recent advances in cell-based therapies for lung diseases provide a platform for development of new therapeutic approaches to acute lung diseases and critical illnesses and possibly also for chronic inflammatory pulmonary conditions including COPD [2, 3]. Mesenchymal stromal cell (MSC)-based therapies have shown promise in a range of pre-clinical lung injury models, including those for COPD [4], due to their immunomodulatory properties (reviewed in 4). However, while systemic MSC administration has proven safe and well-tolerated in clinical investigations, there has been no clear evidence of efficacy to date in a spectrum of lung disease patients studied including those with COPD [5–7]. We previously reported results of a placebo-controlled trial [NCT00683722] of systemic infusions of bone marrow-derived allogeneic MSCs in patients with moderate to severe COPD that included 62 patients with moderate or severe COPD (GOLD spirometry stage 2 or stage 3) randomized at six participating sites to double-blinded intravenous infusions of remestemcel-L, formerly Prochymal™, (10^8^ cells/ infusion) or vehicle control [8]. All subjects received four monthly infusions and were followed for two years after the first infusion. Endpoints included safety assessments, pulmonary function testing, six-minute walk distance (6MWD), frequency of subsequent COPD exacerbations, and patient reported outcome surveys (Borg dyspnea score, St. George Respiratory Questionnaire). Circulating inflammatory biomarkers, including C-reactive protein (CRP), were assessed at baseline and over time. Remestemcel-L was well tolerated with no infusional toxicities and no attributable serious adverse events causally related to treatment as assessed by study investigators. However, there were no statistically significant differences between remestemcel-L and placebo-treated subjects in the pulmonary function, functional capacity, or patient-reported outcome measures. One novel observation was that, in patients with elevated baseline CRP levels at study entry, a statistically significant CRP decrease was observed over the initial months of the study in patients receiving remestemcel-L compared to placebo, and a non-significant trend persisted over the two year observation period.

Since that study was reported, there has been growing appreciation that the function of systemically administered MSCs can be significantly affected by the inflammatory environment they encounter [9–13]. This can manifest as a change in the portfolio of secreted mediators and subsequent downstream actions on relevant inflammatory cells, including macrophages and neutrophils, important in COPD pathogenesis. Circulating CRP is frequently elevated in COPD patients and is associated with higher mortality, worse outcomes after a COPD exacerbation, and for a higher rate of hospital readmission [14–17]. Although patients with any recent exacerbations were excluded from participating in the study, there was a subgroup with elevated circulating CRP levels at baseline, suggestive of a more inflammatory and exacerbation-prone phenotype. We therefore performed a graded post-hoc analysis of functional outcomes from the original investigation based on stratification of circulating CRP levels.

## Methods

Complete study methods including inclusion/exclusion criteria and study assessments are described in detail in the original report [8]. In brief, 62 patients with moderate to severe COPD were randomized to receive 4 monthly infusions of either remestemcel-L or of vehicle control (placebo). The patients were subsequently followed for a 2 year period for safety and potential efficacy. Additional evaluations included measure of circulating mediators including C-reactive protein (CRP). The protocol was approved by the institutional review board at each participating center and written informed consent was obtained from each participant. For the current post-hoc analyses, the full sample of patients who participated in the original study (receiving all four monthly infusions) was stratified into those with serum CRP levels of either ≥ 4 mg/L or < 4 mg/L at study entry (baseline). This cut-point was determined empirically based on an observation in the primary paper that patients with CRP levels ≥ 4 mg/L (n = 29) at baseline showed a statistically significant decrease in CRP at 30 days after the first infusion in MSC-treated patients [8]. Additional sensitivity analyses were applied to evaluate lower cut-points; baseline CRP ≥ 2 (n = 35) or ≥ 3 (n = 42) mg/L). Differences between patients receiving remestemcel-L or placebo in each stratified cohort were assessed with respect to changes from baseline in the pulmonary, functional, and patient-reported outcome variables: forced expiratory volume in 1 s (FEV_1_), forced vital capacity (FVC), total lung capacity (TLC), diffusing capacity for carbon monoxide (D_L_CO), 6 min walk distance (6MWD), number of COPD exacerbations over the 2-year observation period, Borg dyspnea score, and the St. George Respiratory Questionnaire (SGRQ) total score. The study treatment infusions were administered on study days 0 (baseline), 30, 60 and 90. Efficacy assessments were performed at study days 0, 10, 30, 60, 90, 120, 150, 180, 360 and 720 (TLC only at days 0 and 180). The frequency of assessments for each efficacy variable are described in detail in the original publication. Changes over time in levels of circulating CRP were also re-assessed in the stratified cohorts at each study visit. For clarification: all circulating CRP levels were measured in the same centralized laboratory using a high sensitivity assay [8].

Changes from baseline over time in spirometric variables (FEV_1_, FVC) and in 6MWD were assessed using the mixed model for repeated measures analysis of variance (MMRM) to assess overall treatment effects across all visits as well as at each visit separately. The imputation used by the procedure is based on assumption of the missing at random (MAR). An unstructured covariance matrix was used for the MMRM analysis, unless the model does not converge, in which case the compound symmetry covariance matrix was used [18]. A sensitivity test of the difference between remestemcel-L and placebo at day 120 was conducted using Student t-tests for independent groups. Day 120 was chosen as 30 days after last infusion was anticipated to be sufficient time to see effect of treatment. Differences between groups in the proportion of subjects with none/one or two or more COPD exacerbations were tested using a Chi-square test for differences between two proportions. All statistical analyses were performed using two-sided hypothesis tests at the 0.05 level of significance. Data were analyzed using SAS release 9.4 and GraphPad Prism version 8.3.

## Results

Baseline demographic and disease characteristics for subjects with baseline CRP ≥ 4 mg/L and < 4 mg/L are shown in Table 1. There were no significant differences between treatment groups in any of the baseline variables based on this stratification. Mean baseline CRP levels (± SD) in subjects with baseline CRP ≥ 4 mg/L were 16.3 ± 17.2 and 10.3 ± 9.17, and median CRP levels were 10.5 and 7.8 respectively in the remestemcel-L (N = 12) and placebo (N = 17) groups,; in subjects with baseline CRP < 4 mg/L mean baseline CRP values were 1.9 ± 1.2 and 1.7 ± 0.95 mg/L and median values were 1.4 and 1.7 in the remestemcel-L (N = 18) and placebo (N = 15) groups, respectively. The mean CRP values differ ~ seven-fold between CRP strata**.** Within each CRP stratum, baseline CRP levels were similar in the remestemcel-L and placebo groups.

**Table 1:** Demographic and disease characteristics: A. subjects with baseline CRP ≥ 4 mg/L and B. subjects with baseline CRP < 4 mg/L

A. Subjects with baseline CRP ≥ 4 mg/L		
	Placebo (n = 17)	Remestemcel-L (n = 12)
*Demographic characteristics*		
Age, years^a^	65.0 (7.7)	69.3 (5.9)
Gender, n (%)		
Male	8 (47%)	7 (58%)
Female	9 (53%)	5 (42%)
Race, n (%)		
Caucasian	15 (88%)	11 (92%)
Black	2 (12%)	1 (8%)
Current smokers, n (%)	5 (29%)	2 (17%)
Pack-years	64.8 (19.0)	54.2 (17.2)
*Disease characteristics*		
Years since COPD diagnosis	8.9 (6.4)	6.6 (3.3)
Severe disease, n (%)	12 (70.6%)	8 (66.7%)
FEV_1_ (% predicted)	45.6 (12.7)	45.6 (14.8)
FVC (% predicted)	78.9 (18.9)	74.6 (11.6)
FEV_1_/FVC	0.442 (0.1)	0.451 (0.12)
6MWD (m)	317.7 (95.0)	280.6 (117.8)

B. Subjects baseline CRP < 4 mg/L		
	Placebo (n = 15)	Remestemcel-L (n = 18)
*Demographic characteristics*		
Age, years ^a^	62.9 (10.0)	67.3 (8.5)
Gender, n (%)		
Male	10 (66.7)	11 (61.1)
Female	5 (33.3)	7 (38.9)
Race, n (%)		
Caucasian	1313 (86.7)	18 (100)
Black	1 (6.7)	
Asian	1 (6.7)	
Current smokers, n (%)	7 (46.67)	3 (16.7)
Pack-years	50.9 (23.4)	57.1 (23.7)
*Disease characteristics*		
Years since COPD diagnosis	6.2 (6.2)	9.2 (4.8)
Severe disease, n (%)	9 (60.0)	12 (66.7)
FEV_1_ (% predicted)	47.3 (12.9)	44.8 (12.6)
FVC (% predicted)	78.9 (18.9)	74.6 (11.6)
FEV_1_/FVC	0.49 (0.15)	0.43 (0.08)
6MWD (m)	372.5 (91.1)	336.2 (103.5)

*CRP* C-reactive protein, *FEV*_*1*_ forced expiratory volume–one second, *FVC* forced vital capacity, *6MWD* 6 min walk distance

^a^Values are mean (SD) unless otherwise noted

**Fig. 1:**
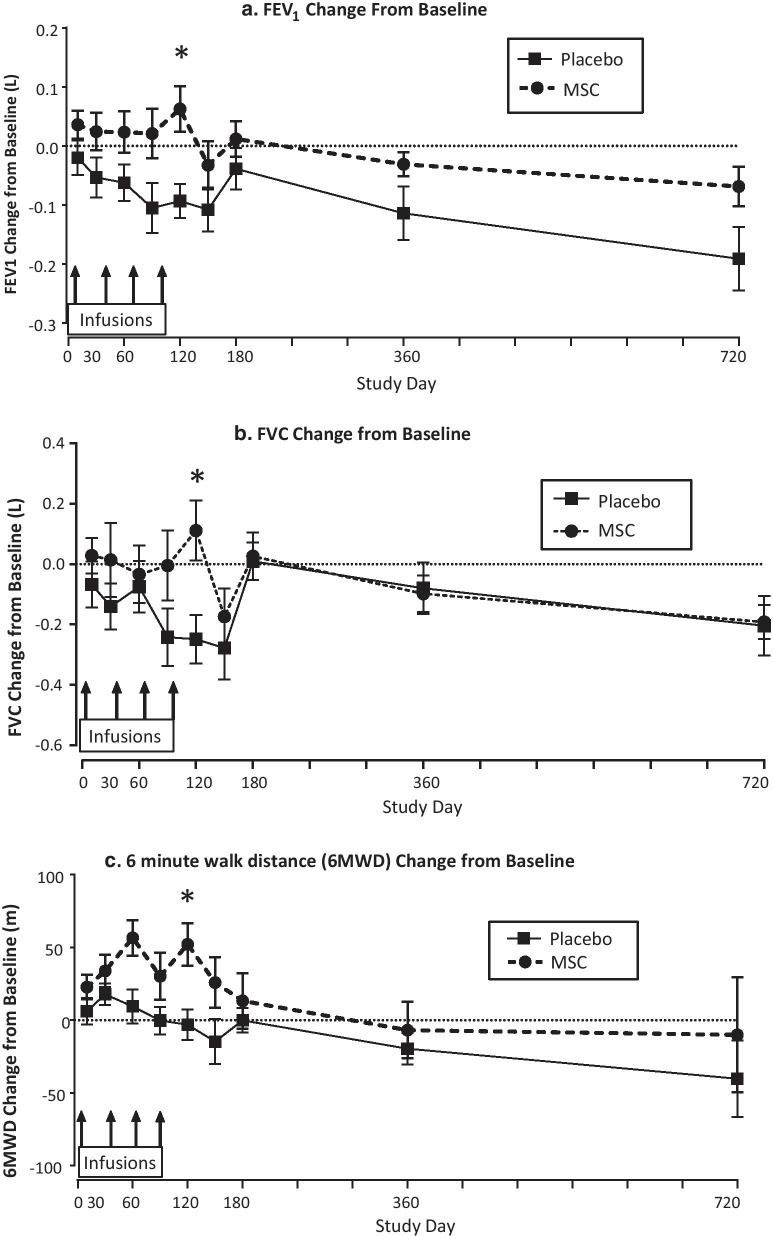
Changes from baseline in pulmonary function and functional performance from baseline in subjects with baseline CRP > 4 mg/L. Overall treatment effect across all visits were assessed by mixed model with repeated measures (MMRM). Simple differences of treatment by visit are assessed by least squares means using MMRM**.** Values at each timepoint are means + SEM. *Signifies P values < 0.05. **a** FEV_1_ (forced expiratory volume:) P = 0.003 overall by MMRM across all visits and P = 0.015 at day 120. **b** FVC (forced vital capacity): P = 0.19 overall by MMRM across all visits and P = 0.005 at day 120. **c **6MWD (six-minute walk distance): P = 0. 0006 overall by MMRM across all visits and P = 0.004 at day 120

**Fig. 2:**
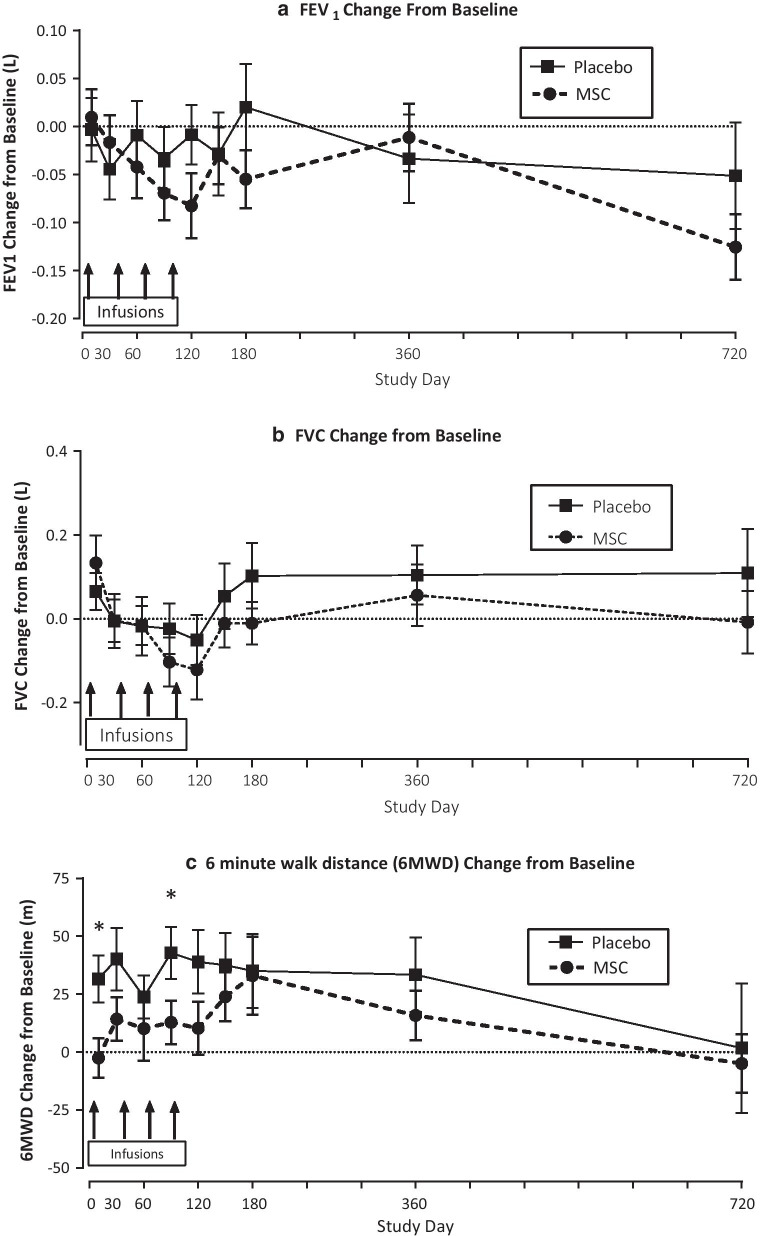
Changes from baseline in pulmonary function and functional performance from baseline in subjects with baseline CRP < 4 mg/L. Overall treatment effect across all visits were assessed by mixed model with repeated measures (MMRM). Simple differences of treatment by visit are assessed by least squares means using MMRM**.** Values at each timepoint are means + SEM. *Signifies P values < 0.05. **a** FEV_1_ (forced expiratory volume:) P = ns overall by MMRM across all visits. **b** FVC (forced vital capacity): P = ns overall by MMRM across all visits. **c** 6MWD (six-minute walk distance): P = ns overall by MMRM across all visits and P < 0.05 at day 10 and day 90

**Fig. 3:**
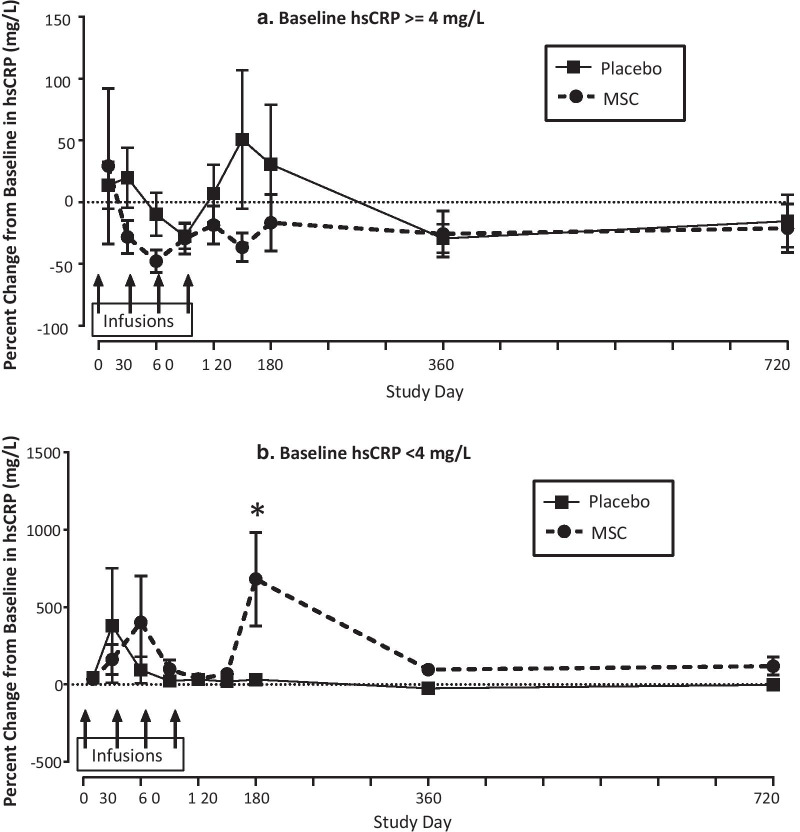
Percent change from baseline in C-reactive protein (CRP) in subjects with baseline CRP ≥ 4 mg/L (panel **a**) and < 4 mg/L (panel **b**). Overall treatment effect across all visits using mixed model with repeated measures (MMRM). There were no significant differences between treatment groups overall or at any individual timepoint except as noted. Values are means + SEM. *Signifies P value < 0.05

Changes over the 2-year study period in FEV_1_, FVC, and 6MWD, and in circulating CRP levels in subjects with baseline CRP ≥ 4 mg/L in those receiving remestemcel-L vs. placebo are shown in Figs. 1 and 3, respectively. Remestemcel-L-treated patients had significant improvements or stabilization of both FEV_1_ and 6MWD as early as 10 days after the first of four monthly study drug infusions. The improvement in FEV_1_ (Fig. 1a) relative to placebo was significant over the 2-year study period as assessed by the mixed model with repeated measures (P = 0.003) with specific significant differences observed at day 120 as assessed by the least squares mean at this timepoint (P = 0.015). Improvement in 6MWD (Fig. 1c) persisted through 120 days and a significant trajectory compared to placebo was evident over the entire 2-year study period (P = 0.006 overall; P = 0.004 at day 120). In contrast, placebo-treated patients showed a progressive overall decrease in both FEV_1_ and 6MWD over the 2-year study period (Fig. 1). FVC stabilized over the first 120 days in remestemcel-L-treated as compared to progressive decline in placebo treated patients (Fig. 1b). Although the overall change from baseline by MMRM was not significant for FVC (P = 0.19), isolated significance compared to placebo was observed at day 120 (P = 0.005.) However, after 180 days, both groups showed similar progressive declines. There were no significant changes in total lung capacity at day 180 or in D_L_CO in patients with baseline CRP ≥ 4 mg/Lin either the remestemcel-L or placebo groups. In contrast to the effects noted in patients with baseline CRP ≥ 4, in patients with baseline CRP < 4 mg/L overall there were no significant differences overall or at any visit between treatment groups in FEV_1_ and FVC, and no significant differences in 6MWD except at day 10 and day 90, which favored placebo (Fig. 2).

For patients with baseline CRP ≥ 4 mg/L there was a trend towards reductions in CRP levels over the first 180 days in the remestemcel-L-treated compared with placebo-treated subjects (Fig. 3a). However, these did not correlate with the observed changes in FEV_1_, FVC, or 6MWD. For patients with CRP < 4 mg/L there was no difference between treatment groups in CRP levels except at Day 180 in favor of the placebo group (Fig. 3b). There were no significant changes or differences in the number of COPD exacerbations, Borg dyspnea score, or SGRQ results between patients treated with remestemcel-L vs. placebo in either CRP stratum. To explore further the influence of circulating CRP levels on FEV_1_, FVC, and 6MWD, patients were further stratified into those with levels of either ≥ or < than 3 or 2 mg/L at baseline. Values were assessed on Day 120, the time point at which the greatest differences between MSC vs. placebo treated patients were observed when stratified for CRP levels ≥ or < 4 mg/L. Notably, improvements in FEV_1_, FVC, and 6MWD in remestemcel-L- vs placebo-treated patients were also observed in those with baseline CRP ≥ 3 mg/dl (Table 2). Comparative improvements in FVC in remestemcel-L- vs placebo-treated patients were further observed in those with baseline CRP ≥ 2 mg/dl while those for FEV_1_ and 6MWD were no longer significant (p < 0.055 and p < 0.159, respectively). There was no change in number of COPD exacerbations over the 2 year study period between remestemcel-L- vs placebo-treated patients when stratified for CRP ≥ or < 4 mg/dl (Table 3).

**Table 2:** Day 120 efficacy analysis by baseline CRP subgroups (sensitivity analysis)

	Placebo	MSC	Difference	P-value^a^
Subgroup/parameter				
*Baseline CRP ≥ 4 mg/L*	*N = 17*	*N = 12*		
Mean change from baseline:				
FEV_1_ (L)	− 0.094	0.063	0.156	0.003
FVC (L)	− 0.249	0.111	0.360	0.009
6MWD (m)	− 3.118	52.167	55.284	0.004
*Baseline CRP < 4 mg/L*	*N = 15*	*N = 18*		
Mean change from baseline:				
FEV_1_ (L)	− 0.009	− 0.083	− 0.074	0.123
FVC (L)	− 0.051	− 0.122	− 0.071	0.461
6MWD (m)	38.933	10.333	− 28.600	0.116
*Baseline CRP ≥ 3 mg/L*	*N = 21*	*N = 14*		
Mean change from baseline:				
FEV_1_ (L)	− 0.074	0.051	0.125	0.009
FVC (L)	− 0.217	0.109	0.326	0.006
6MWD (m)	5.952	42.571	36.619	0.040
*Baseline CRP < 3 mg/L*	*N = 11*	*N = 16*		
Mean change from baseline:				
FEV_1_ (L)	− 0.015	− 0.091	− 0.076	0.165
FVC (L)	− 0.041	− 0.149	− 0.109	0.333
6MWD (m)	36.909	13.500	− 23.409	0.270
*Baseline CRP ≥ 2 mg/L*	*N = 23*	*N = 19*		
Mean change from baseline:				
FEV_1_ (L)	− 0.073	0.009	0.083	0.055
FVC (L)	− 0.199	0.042	0.241	0.022
6MWD (m)	8.391	31.632	23.241	0.159
*Baseline CRP < 2 mg/L*	*N = 9*	*N = 11*		
Mean change from baseline:				
FEV_1_ (L)	− 0.004	− 0.084	− 0.079	0.235
FVC (L)	− 0.047	− 0.150	− 0.103	0.435
6MWD (m)	37.556	19.182	− 18.374	0.444

*CRP* C-reactive protein, *FEV*_*1*_ forced expiratory volume—one second, *FVC *forced vital capacity, *6MWD* 6-min walk distance

^a^P-value for difference between placebo and remestemcel-L groups by Student t-test for independent groups

**Table 3:** Number (percent) of COPD exacerbations per patient by treatment group for subjects with baseline CRP ≥ 4 mg/L and < 4 mg/L

COPD exacerbations per patient		0 or 1	≤ 2
Baseline COPD ≥ 4 mg/L	Placebo N = 17	11 (65)	6 (35)
	Remestemcel-L N = 12	10 (83)	2 (17)
Baseline COPD < 4 mg/L	Placebo N = 15	13 (87)	2 (13)
	Remestemcel-L N = 18	12 (67)	6 (33)

*COPD* chronic obstructive lung disease, *CRP* C-reactive protein

## Discussion

Post-hoc, hypothesis-generating analyses of a previously conducted investigation of systemic MSC administration in patients with moderate-severe COPD have demonstrated previously unrecognized efficacy in those with more systemic inflammation at study entry. Importantly, beneficial effects over time were observed in the clinically relevant functional outcomes FEV_1_, FVC, and 6MWD. Notably, at 120 days, the magnitude of these differences exceeded the meaningful clinically important differences for both FEV_1_ and 6MWD and thus have significant clinical implications. [18, 19] There was a corresponding, albeit non-significant, trend towards decrease in systemic inflammation as assessed by serial measurements of circulating CRP levels in remestemcel-L treated patients but this, however, did not correlate with changes in the functional outcomes. Improvements in these outcomes also appeared to hold when patients were stratified for lower levels of systemic inflammation.

The biological rationale for investigation of potential salutary effects of MSCs in COPD is based on the premise that secretion of multiple paracrine factors including anti-inflammatory cytokines and growth factors that thereby facilitate tissue repair may counter or potentially even reverse chronic inflammation and lung destruction. However, studies to date of MSC administration in COPD patients have consistently demonstrated safety but not efficacy [5–8]. Other studies have further demonstrated safety but were not designed to assess efficacy when MSCs were administered to study effects on systemic immune responses in COPD patients [20]. A recent phase 1 study assessing safety efficacy of MSC administration for potential treatment of inflammation resulting from endobronchial valve placement in COPD patients was not designed to assess specific effects on COPD clinical course [21]. However, this study also demonstrated a decrease in circulating CRP in patients who received MSCs. These results are in contradistinction to a body of literature in pre-clinical models of emphysema, including those resulting from elastase, papain, and cigarette-smoke induced inflammation and damage, in which MSC administration may have beneficial effects [5, 22–44]. Acknowledging the strengths and imitations of each of these models for fidelity to human disease, a variety of mechanisms have been postulated for MSC actions. Many of these focus on disruption of inflammatory pathways activated or provoked in the different models and highlight increasing recognition that MSCs may have better potential therapeutic effects in more inflammatory lung environments [2]. Notably, MSCs are increasingly recognized to sense the inflammatory environment through damage- and pathogen-associated molecular pathogen receptors, e.g. Toll-Like receptors, and respond by releasing specific sets of anti-inflammatory cytokines, anti-bacterial peptides, extracellular vesicles containing anti-inflammatory miRNAs, mitochondria, and other potential mediators. [4, 9–13, 44–49].

As such, we postulate that a subset of patients in the COPD spectrum may be more likely to respond to MSC-based administration, i.e., those with more pronounced chronic inflammation. This has not as yet been directly or prospectively addressed in clinical investigations to date. However, the findings from the post-hoc analyses presented in the current analyses are both mechanistically hypothesis generating and also powerful stimuli to prospectively re-assess potential efficacies of MSC-based cell therapies in carefully stratified patient groups. Accordingly, future studies may focus on patients with high levels of circulating inflammatory cytokines including IL-6, IL-1 and TNF-α as well as CRP to investigate further the clinical efficacy and impact of inflammatory cytokines on cell-based therapies. In the primary analysis of this study, levels of inflammatory cytokines other than for CRP were below the level of detection in most subjects, perhaps due to assay methods that lacked sufficient sensitivity, and high sensitivity CRP will need to be further assessed in future studies [8]. Limitations of this report include the post-hoc nature of the analysis, the lack of data regarding other inflammatory cytokines to confirm the CRP observations and the relatively small sample size. Further, as documented in the original report, review of patients’ diaries kept during the investigation revealed that reliever/rescue medication use was not systematically recorded and it was not possible to do the planned analyses on home medication use including home oxygen. These deserve re-investigation in future prospective studies as will a wider range of inflammatory markers including, for example, circulating receptor for advanced glycation endproducts (RAGE). A review of corticosteroid use revealed that only a small number of patients were on these at study entry, some for conditions other than COPD. This is a parameter that will be investigated further in prospective studies. Further, other conditions may also contribute to fluctuations in CRP and may thus explain the observed variability. Knowledge of how MSCs are acting in different disease environments is also evolving including further appreciation of host responses to systemic administration of allogeneic MSCs [9, 50–52]. Nonetheless, these findings are suggestive and hypothesis-generating for confirmation in future studies.

## Conclusion

In summary, stratification of COPD patients by elevated baseline CRP level identified patients that responded to remestemcel-L treatment with improvements in pulmonary and overall physical function. A trend for an association between highest CRP levels and degree of clinical response suggests that the inflammatory component of COPD may amplify potential beneficial immunomodulatory effects of remestemcel-L administration in COPD. Future studies will be needed to investigate this in greater detail.

## Data Availability

The datasets used and/or analysed during the current study were initially published in Weiss et al., A placebo-controlled, randomized trial of mesenchymal stem cells in COPD. Chest. 2013;143(6):1590–8 and are available from the corresponding author on reasonable request.
